# Regulation of Satellite Cell Function in Sarcopenia

**DOI:** 10.3389/fnagi.2014.00246

**Published:** 2014-09-22

**Authors:** Stephen E. Alway, Matthew J. Myers, Junaith S. Mohamed

**Affiliations:** ^1^Laboratory of Muscle Biology and Sarcopenia, Department of Exercise Physiology, West Virginia University School of Medicine, Morgantown, WV, USA; ^2^West Virginia Clinical and Translational Science Institute, Morgantown, WV, USA; ^3^Center for Cardiovascular and Respiratory Sciences, Morgantown, WV, USA

**Keywords:** aging, oxidative stress, apoptosis, rehabilitation, injury, disuse atrophy

## Abstract

The mechanisms contributing to sarcopenia include reduced satellite cell (myogenic stem cell) function that is impacted by the environment (niche) of these cells. Satellite cell function is affected by oxidative stress, which is elevated in aged muscles, and this along with changes in largely unknown systemic factors, likely contribute to the manner in which satellite cells respond to stressors such as exercise, disuse, or rehabilitation in sarcopenic muscles. Nutritional intervention provides one therapeutic strategy to improve the satellite cell niche and systemic factors, with the goal of improving satellite cell function in aging muscles. Although many elderly persons consume various nutraceuticals with the hope of improving health, most of these compounds have not been thoroughly tested, and the impacts that they might have on sarcopenia and satellite cell function are not clear. This review discusses data pertaining to the satellite cell responses and function in aging skeletal muscle, and the impact that three compounds: resveratrol, green tea catechins, and β-Hydroxy-β-methylbutyrate have on regulating satellite cell function and therefore contributing to reducing sarcopenia or improving muscle mass after disuse in aging. The data suggest that these nutraceutical compounds improve satellite cell function during rehabilitative loading in animal models of aging after disuse (i.e., muscle regeneration). While these compounds have not been rigorously tested in humans, the data from animal models of aging provide a strong basis for conducting additional focused work to determine if these or other nutraceuticals can offset the muscle losses, or improve regeneration in sarcopenic muscles of older humans via improving satellite cell function.

## Introduction

Sarcopenia is the age-associated reduction in muscle mass and function (Evans, 1995; Kim and Choi, 2013), which is particularly severe after the seventh decade of life (Dutta et al., 1997). Sarcopenia increases the susceptibility to muscle injury (Faulkner et al., 1995), serious falls (Tinetti, 2001), obesity (Stenholm et al., 2008), and diabetes (Kim et al., 2010; Ghosh et al., 2014). Furthermore, the deleterious effects of extended muscle disuse (e.g., prolonged bed rest in the elderly) on muscle mass, strength, and function is exacerbated with sarcopenia (Suetta et al., 2009; Marzetti et al., 2010; Hao et al., 2011; Calvani et al., 2013; Alway et al., 2014a). As a result, it is important to identify strategies that could slow or reverse sarcopenia. One area that has attracted recent attention is the area of myogenic stem cells or satellite cells, as a means to improve regeneration of old muscles and to offset the negative consequences of sarcopenia.

## Mechanisms That may Contribute to Sarcopenia and Lower the Ability to Reverse Atrophy in Aging

To mount an effective therapeutic strategy to treat sarcopenia, it becomes necessary to understand the components that contribute to this pathogenesis. While the mechanisms responsible for sarcopenia are not well understood, there are likely several factors that contribute to muscle loss in aging. These include but may not be limited to: reduced protein synthesis (Dickinson et al., 2013; Churchward-Venne et al., 2014), declines in neural function (Drey et al., 2013; Kwan, 2013; Mosole et al., 2014), hormonal deficits (Michalakis et al., 2013), chronic inflammation (Lee et al., 2007; Degens, 2010; Mavros et al., 2014), oxidative stress (Hiona and Leeuwenburgh, 2008; Jackson et al., 2010; Armand et al., 2011; Marzetti et al., 2013; Sullivan-Gunn and Lewandowski, 2013), loss of mitochondrial function (Chabi et al., 2008; Ljubicic et al., 2009; Calvani et al., 2013; Marzetti et al., 2013), inappropriate signaling in muscle due at least in part to inadequate nutrition (Burgos, 2012; Ghosh et al., 2014; Welch, 2014; Welch et al., 2014), nuclear apoptosis (Sjostrom et al., 1992; Alway et al., 2002, 2011; Leeuwenburgh, 2003; Dupont-Versteegden, 2005; Alway and Siu, 2008; Chabi et al., 2008), and reduced satellite cell function (Conboy and Rando, 2005; Snijders et al., 2009; Barberi et al., 2013). This review will focus on the potential impact that mediation of satellite cell function has in aging skeletal muscle.

## Satellite Cell Biology

Satellite cells are a heterogeneous collection of adult muscle stem cells that are normally quiescent. They were first identified more than 50 years ago as a unique population of nuclei that were “sandwiched” between the sarcolemma and the basement membrane of the muscle fiber (Mauro, 1961). Utilization of unique cell surface markers and methods to identify satellite cell proliferation and differentiation have provided evidence to show that this cell is critically important in muscle growth and repair as well as the processes of adaptation to stresses including exercise, disease, injury, and aging. Satellite cell progression from proliferation through differentiation of their daughter cells is tightly regulated by muscle transcription factors. Adult quiescent satellite cells express the paired homeobox transcription factor Pax7 (Seale et al., 2000, 2004). Another transcription factor, Pax3, a paralog of Pax7, is also expressed in a subset of satellite cells of some but not all muscles (Relaix et al., 2006; Buckingham and Relaix, 2007; Day et al., 2007; Yablonka-Reuveni et al., 2008). Nevertheless, Pax7 appears to be necessary in satellite cells after birth as Pax7-null mice are viable but lack any functional satellite cells (Kuang et al., 2006; Seale et al., 2000; Seale et al., 2004).

**Figure 1:**
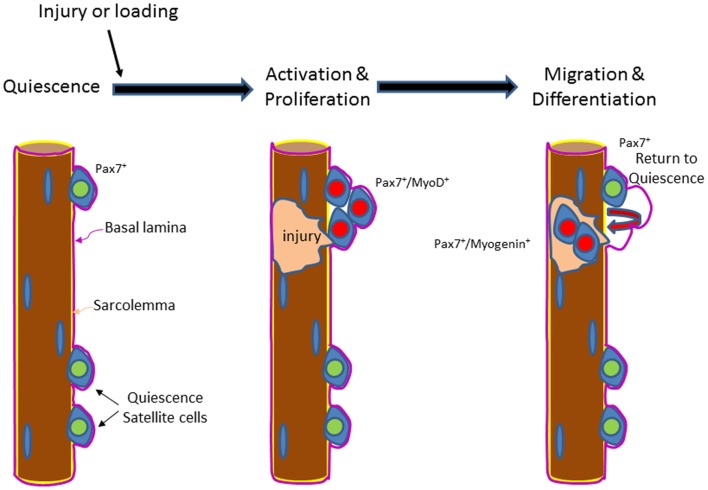
**Illustration of the activation and the differentiation of satellite cells in muscles of young or old hosts**. Satellite cells are positioned anatomically between the basal lamina (purple) and the sarcolemma (yellow). Satellite cells express Pax7 in the quiescent (green) state. Upon injury or loading, satellite cells are activated and proliferate (red) and express Pax7 followed by both Pax7 and MyoD. Some of the proliferated daughter cells from the original satellite cells leave their position and pass through the sarcolemma and migrate to the site of injury, where they fuse with the existing fiber and engage in fiber repair and/or contribute to fiber growth/hypertrophy. In aging and sarcopenic muscles, satellite cell proliferation and/or differentiation may be impaired, which contribute to a lower regenerative potential.

Under basal conditions, adult satellite cells remain quiescent and reside (relatively) dormant within their niche adjacent to the myofiber (Schultz et al., 1978) (Figure 1). While satellite cells might be exposed to the changing cellular niche, they do not become activated until a major insult or stress (e.g., exercise loading) occurs. In response to injury, satellite cells proliferate and their Pax7-positive daughter cells either differentiate, by migrating through the sarcolemma and fusing with existing muscle fibers (Figure 1) during the growth and regeneration of muscle (Moss and Leblond, 1971; von et al., 2013), or they commit to a program of self-renew (Schmalbruch and Lewis, 2000; Collins et al., 2005). Myogenic regulator factor (MRF) genes provide myogenic specificity for activated satellite cells. The MRFs include myogenic differentiation 1 protein (MyoD), myogenic factor 5 (Myf5), myogenin, and muscle-specific regulatory factor 4 (Mrf4). Myf5 and/or MyoD expression are quickly increased at the point of satellite cell activation (Cornelison and Wold, 1997). Pax7 regulates Myf5 and MyoD expression levels (Parise et al., 2008; Rudnicki et al., 2008) in satellite cells. It is necessary for Pax7 to be down-regulated prior to terminal differentiation of the satellite cell derived daughter cells (Olguin and Olwin, 2004; Olguin et al., 2007). The satellite cell pool is repopulated by the fraction of activated satellite cells that maintain a high level of Pax7.

A number of studies using different Pax7 ablation strategies in mouse muscles, have clearly shown that satellite cells are indispensable for muscle regeneration (Lepper et al., 2011; Murphy et al., 2011; Sambasivan et al., 2011). Furthermore, when satellite cells are absent, injured skeletal muscle does not regenerate or regenerate very poorly in response to muscle injury (Seale and Rudnicki, 2000; Seale et al., 2000; Oustanina et al., 2004; Kuang et al., 2006). To make it worse, inflammatory and adipogenic cells replace injured regions of contractile tissue in muscles from Pax7-null animals (Sambasivan et al., 2011), and the increase in non-contractile tissue in repair or growth in the absence of Pax7 decreases the specific tension (force per cross sectional area) potential in skeletal muscle (Fry et al., 2014). It is also important to note that non-myogenic stem cells are unable to repair the injured muscle, suggesting that satellite cells are essential for skeletal muscle regeneration (Sambasivan et al., 2011). Furthermore, while some muscle hypertrophy that is induced via overload appears possible in the absence of satellite cells, long-term muscle adaptation to overload is diminished, as seen by smaller muscle fibers and a lower muscle mass and muscle strength. Thus, satellite cells are important not only in muscle repair but also in regulating muscle adaptations to hypertrophic growth (Fry et al., 2014). Thus, it is possible, or even likely that a diminished function in satellite cells plays an important role in mediating the long-term muscle reductions with sarcopenia.

## Satellite Cell Function in Aging

The potential mechanisms involved in the reduction of skeletal muscle mass during sarcopenia converge on satellite cells, and together they contribute to failure of satellite cells to replace and repair damaged muscle fibers (Jang et al., 2011; Garcia-Prat et al., 2013; Wang et al., 2013; Sousa-Victor et al., 2014). The lower regenerative potential of aged muscles is correlated nicely with the decline in satellite cell function (Jang et al., 2011; Chakkalakal and Brack, 2012; Collins-Hooper et al., 2012; Bernet et al., 2014) and a reduction in the Pax7 pool of myogenic stem cells (Collins et al., 2007). For example, the proliferation and differentiation potentials of satellite cells in both mammals and non-mammals are reduced with increasing age (Bortoli et al., 2005; Velleman et al., 2010; Barberi et al., 2013; Harthan et al., 2013). In addition, recent evidence by Sousa-Victor et al. (2014) suggest that a large portion of the aged (geriatric) satellite cells switch from the reversible quiescent state to a senescence state, which prevents proliferation and renewal of the satellite cell pool. Thus, this loss of satellite cell function likely contributes strongly to the reduced ability to repair or replace muscle that is lost in sarcopenia. Although satellite cells in the aged niche are not proliferative, they do express Sprouty1 (*spry1*), an inhibitor of fibroblast growth factor (FGF) signaling (Chakkalakal et al., 2012). It is thought that increasing FGF signaling in aged satellite cells under basal conditions by down regulating *spry1* would result in a loss of quiescence (Chakkalakal et al., 2012). Thus, aged satellite cells may actively promote quiescence through regulating *spry1* in their own niche, thereby making it more difficult to activate these cells for growth or repair.

Moreover, satellite cell content has been reported to decrease in muscles of old humans and animals as compared to their younger counter parts (Day et al., 2010; Verdijk et al., 2012, 2014). Furthermore, there is evidence that a decline in satellite cell number contributes to muscle fiber atrophy (Brack et al., 2005). Nevertheless, some studies have not found a loss of satellite cells in old muscles as compared to muscles from young animals (van der Meer et al., 2011b), but this is complicated by the fact that although muscle mass/bodyweight was lower in the old animals, the absolute muscle mass was similar in young and old animals.

Whether satellite cell number is lost or not, it appears more clear that satellite cell function is reduced in aging. However, it is likely that an important cause for reduced satellite cell function in aging may be a result of altered systemic factors that influence and/or regulate satellite cell activity and differentiation. Notably, important observations from Rando and colleagues using parabiotic pairs have shown that the regenerative potential of satellite cells can be improved in muscles from aged mice that share the circulation with young mice (Conboy and Rando, 2005; Conboy et al., 2005). Reductions in Notch signaling in muscles of aged rodents lead to a reduced satellite cell proliferation and an inability to produce myoblasts in response to muscle injury. In addition, restoring circulating levels of protein growth differentiation factor 11 (GDF11) in old mice has recently been shown to improve satellite cell and muscle function (Sinha et al., 2014). Other factors contributing to sarcopenia potentially through their actions on satellite cells could involve reduced IGF-I (Harridge, 2003), inflammation and pro-inflammatory cytokines (Degens, 2010), and altered muscle metabolism (Jang et al., 2011).

Although satellite cells appear to have important roles in regeneration of old or young muscles, their involvement in regulating muscle mass in response to atrophic or hypertrophic stimuli is quite complex. For example, rapid muscle loss occurring from denervation has been reported to result in a transient increase in satellite cells in muscles of old rats within 1 week after denervation (van der Meer et al., 2011b), presumably in an attempt to improve the transcriptional control of muscle proteins during this rapid period of atrophy. However, satellite cell numbers then decreased in old muscle in subsequent denervation from 2 to 4 weeks (although satellite cells/muscle cross sectional area were constant during this time) (van der Meer et al., 2011b). In contrast, muscles in young animals had an increase in satellite cell numbers over 4 weeks of denervation (van der Meer et al., 2011b), yet the increase in satellite cell numbers was unable to prevent muscle atrophy (van der Meer et al., 2011b). Clearly, there are age-induced differences in the responses of satellite cells to this atrophy stimulus by denervation, yet simply having the potential for greater transcriptional control by having more satellite cells and their daughter cells, fails to prevent the rapid muscle fiber atrophy caused by denervation.

The role that satellite cell number has in muscle growth is also complex. For example, there is evidence that at least some degree of hypertrophy can occur without the prerequisite to activate satellite cells to add new nuclei (McCarthy et al., 2011; van der Meer et al., 2011a; Jackson et al., 2012); however, larger fibers in old muscles appear to add more nuclei than smaller fibers in young animals to maintain a relatively constant nuclear domain size (van der Meer et al., 2011b), and more nuclei improve the potential for greater transcriptional control to presumably sustain their new larger muscle fiber size (Carson and Alway, 1996; Alway et al., 2003; van der Meer et al., 2011a). In addition, the extent of hypertrophy is suppressed in models where satellite cells are absent (Fry et al., 2014). Thus, satellite cells may have an important role in long-term modulating of muscle fiber size, but at least in some models of muscle wasting, increases in myonuclear number does not guarantee greater fiber sizes in denervation, and losses in satellite cells over time, follows, and does not determine, the reductions in muscle fiber size (van der Meer et al., 2011a,b). Nevertheless, it is clear that hypertrophic adaptations are suppressed when satellite cells are eliminated, and therefore, it is likely that satellite cells have a complex modulating effect on muscle mass, and in doing so, impacts muscle function. In addition, we would expect that loss of satellite cells or reduced satellite cell function by whatever means, would diminish the ability for aging muscle to both hypertrophy in response to a growth stimulus, and repair in response to an injury. Clearly, this area requires more work to fully understand the complex nature and responses of satellite cells in muscle remodeling in aging.

## Modulators of Satellite Cells in Sarcopenia

### Metabolic regulation of satellite cell function

Metabolic regulators of the satellite cell niche are likely to be important modulators of satellite cell function. One potential mediator is Sirtuin 1 (Sirt1), a NAD^+^ deacetylase that is activated by caloric restriction (Cohen et al., 2004) and resveratrol (Chen et al., 2009; Price et al., 2012), a compound found in abundance in grape skins and red wine. Sirt1 also works in concert with a number of transcription factors to exert a mostly catabolic effect in cellular metabolism. One subset of these activated transcription factors is the peroxisome proliferator-activated receptor (PPAR) family (Lin et al., 2005), including PPAR γ co-activator 1α (PGC1α). PGC1α is a transcriptional co-activator and a major regulator of mitochondrial biogenesis and metabolism (Spiegelman, 2007; Stepto et al., 2012). In muscle and other cell types, PGC1α regulates the activity of PPAR alpha (PPARα) and PPAR delta (PPARδ). PPARα, is expressed in the heart, liver, and skeletal muscle, regulates mitochondrial biogenesis and fatty acid uptake and oxidation. PPARδ is expressed in the intestines, liver, and skeletal muscle but notably, experiments that have eliminated PPARδ in muscle, have reported a decreased level of satellite cell proliferation, leading to reduced muscle regenerative capacity after injury, further establishing its link to satellite cell function (Angione et al., 2011). PGC1α induced mitochondrial biogenesis appears to be an important component that regulates satellite cell function in regenerating muscle following injury (Duguez et al., 2002). Supporting this finding, short-term caloric restriction has been found to increase satellite cell proliferation in young and old mice, presumably through a Sirt1-PGC1α mechanism (Cerletti et al., 2012). Furthermore, alterations in PGC1α through Sirt1 have been shown to reduce satellite cell-induced muscle regeneration during conditions of muscle wasting (Toledo et al., 2011). Thus, there appears to be a clear link between mitochondrial biogenesis/function and satellite cell function, and increasing mitochondrial function increases satellite cell proliferation in muscle regeneration (Jash and Adhya, 2012). However, the mechanisms that mediate this interaction are less clear and the impact that altering mitochondrial-mediated metabolic function will have on satellite cell function in muscles of old animals is less well known. Furthermore, it is not known if mitochondrially induced satellite cell modulation is fiber-type specific in aged muscles.

### Mitochondrial function and oxidative stress regulate satellite cell function in aging

In addition to metabolic function, mitochondria are key producers of reactive oxygen species (ROS). A low level of ROS is thought to be an important regulator of several cell signal transduction pathways in a variety of cellular functions including muscle (Frey et al., 2006, 2009; Powers et al., 2011). However, excessive ROS levels are believed to be key initiators and mediators of dysfunction in a variety of cells including muscle cells. This includes ROS mediated disruptions in cell signaling, metabolism, transcriptional activity, mitochondrial function, and increased activation of apoptotic pathways (Allen and Tresini, 2000; Marzetti et al., 2013; Sullivan-Gunn and Lewandowski, 2013). For example, aging is associated with excessive ROS levels, which increases mitochondrial damage, and in turn, contributes to mitochondrially mediated apoptotic signaling (Barberi et al., 2013; Bennett et al., 2013; Szczesny et al., 2013; Vasilaki and Jackson, 2013). This suggests that mitochondria might produce high ROS levels in muscle and in activated satellite cells, and this could contribute to impaired satellite cell function (or initiate pathways that could result in satellite cell death). However, this relationship is complex because human muscle satellite cells that were isolated from elderly human vastus lateralis muscles have reduced mitochondrial mass, and lower whole cell ATP levels, but when they were stimulated maximally, they appeared to have normal mitochondrial ATP production, increased mitochondrial membrane potential, and increased superoxide/mitochondrial mass and hydrogen peroxide/mitochondrial mass ratios (Minet and Gaster, 2012). These data suggest that although ROS production was higher in isolated satellite cells from aged muscles, there was not a marked reduction in mitochondrial function of the remaining mitochondria (Minet and Gaster, 2012). Nevertheless, the high basal levels of ROS may provide an unfavorable environment, which can adversely affect satellite cell function and limit muscle repair in aging. One of the possible causes that could exacerbate the effects of ROS on satellite cell function is the decline the antioxidative capacity and the increasing ROS levels of skeletal muscle with increasing age, which diminish satellite cell function (Beccafico et al., 2007). Moreover, the antioxidant activity of catalase and glutathione transferase is reduced in satellite cells derived from elderly subjects as compared to satellite cells isolated from young individuals (Fulle et al., 2005). It is likely that the elevated basal levels of ROS in the satellite cell niche could induce oxidative damage to the quiescent satellite cells, and this damage could negatively affect the satellite cells’ ability to repair aging muscle when they become activated (Fulle et al., 2005).

An alternative negative effect of high ROS levels in aging muscles may be to drive the normal myogenic phenotype of activated satellite cells to that of an adipogenic phenotype. Such a muscle-to-fat transition and satellite cell behavior could explain the increase in inter-muscular adipose deposits that are characterized with many metabolic diseases as well as sarcopenia (Rice et al., 1989; Vettor et al., 2009).

### Exercise regulation of satellite cells in aging muscle

Although exercise or loading can partially rescue the reduction in satellite cell function (Dreyer et al., 2006; Snijders et al., 2009; Verdijk et al., 2009; Shefer et al., 2010), muscle fibers typically do not hypertrophy to the same extent in old animals as compared to young animals, even if the young and old animals receive the same stimulus and this is at least partially attributable to aging-suppressed satellite cell function (Carson et al., 1995; Carson and Alway, 1996; Lowe et al., 1998; Cutlip et al., 2006). Thus, while full reversal of sarcopenia does not appear to be possible, exercise, and overload nevertheless, have been used as a rehabilitative tool to compensate for sarcopenia, and can at least partially reverse the age-imposed decrements in performance. Nevertheless, experiments that have examined the exercise- and loading-associated reduction in muscle mass function in humans, rats, and other animal models of aging, have produced varied results from modest to poor reversal of sarcopenia. For example, 30 days of identical loading conditions resulted in 44% greater muscle mass in young-adult birds, but only an increase in muscle mass of 25% in aged quails (Carson and Alway, 1996). In addition, 14 days of functional overload in the rat plantaris muscle increases muscle weight by 25% in young-adult animals but only by 9% in old rats (Alway et al., 2002, 2005). Furthermore, 28 days of electrical stimulation-induced contractions caused muscle hypertrophy and improved function in young rat dorsi flexor muscles, but no improvement in muscle force or mass were found in old animals (Cutlip et al., 2006; Murlasits et al., 2006).

It is clear that loading types of exercise have profound effects on satellite cell function in muscles of young hosts, largely through activation of various growth factors and cytokines, resulting in increases in muscle protein synthesis, and net muscle protein accretion (Phillips et al., 1997). Furthermore, activation of satellite cells occurs as part of the modulation of exercise-induced adaptation even in acute responses to loading exercise where hypertrophy has not occurred (Joanisse et al., 2013). Specifically, growth factors such as insulin like growth factor-I (IGF-1) (McKay et al., 2008), interleukin-6 (IL-6) cytokine induced signal transducer, and activator of transcription 3 (STAT3) signaling have been shown to occur exclusively in human satellite cells, including their proliferation in response to exercise-induced lengthening injury (Toth et al., 2011). Furthermore, non-injurious running exercise has been shown to increase Wnt signaling and that activation of the canonical Wnt/β-catenin signaling pathway increased the expression of Myf5 and MyoD in satellite cells (Fujimaki et al., 2014). However, given the greater senescent phenotype of satellite cells in aged muscles (Sousa-Victor et al., 2014), it seems likely that exercise would have a greater challenge for activating satellite cells in aged sarcopenic muscles as compared to young-adult muscles. Nevertheless, while aging decreases the satellite cell content in type II fibers of humans (Verdijk et al., 2014), aging appears to delay but not eliminate the activation of satellite cells in muscles of elderly men in response to acute resistance exercise (Snijders et al., 2014a; Verdijk et al., 2014).

While an age-associated reduction in growth differentiation factor 11 (GDF11) has been shown, a recent report indicates that restoring systemic levels of GDF11 in aged muscle improved not only satellite cell derived muscle repair but increased muscle strength, mass, and endurance in aged mice (Sinha et al., 2014). This shows the potential for important interactions between circulating factors and exercise-induced satellite cell function; however, it would seem that exercise alone, without the pharmacological intervention and interaction is insufficient to reverse all of the aging-associated satellite cell function in sarcopenia. Although electrically evoked contractions are not exact duplications of voluntary exercise, most of the cellular signaling pathways are similar whether the contractions are voluntary or evoked. Thus, it is interesting to note that electrically evoked contractions in muscles of elderly subjects, increased the proliferation of satellite cells as indicated by a greater number of N-CAM and Pax7-expressing cells (surface markers of satellite cells), and also increased IGF-I and myostatin, which, were thought to loosely represent markers in the pathway for satellite cell differentiation (Kern et al., 2014). Thus, exercise and models that simulate exercise have profound effects on satellite cell function in aging muscles. One of the challenges in muscle biology is to identify targets and strategies that are likely to maximize the positive benefits of exercise on satellite cell function with the goal to reduce or offset sarcopenia.

### Disuse reduces satellite cell number in aging

Disuse atrophy is caused by mechanical unloading of muscle and this leads to reduced muscle mass. Frequently used models of unloading in humans include casting/immobilization, and a sedentary lifestyle (inactivity), and in rodents, hindlimb suspension, immobilization, and denervation are typically used as models of disuse. Satellite cells are fundamentally involved in skeletal muscle responses to environmental changes that induce atrophy. The area that surrounds the satellite cell (niche) plays an important role in the fate and function of satellite cells (Bentzinger et al., 2013), and therefore, it is not surprising that changes in the muscle environment that occur during disuse can affect the satellite cell niche.

Several studies report that conditions of disuse lead to an elevation in the number of nuclei that have been targeted for apoptosis both inside and outside myofibers (Allen et al., 1997; Vescovo et al., 1998, 2000; Siu and Alway, 2009; Alway et al., 2011; Hao et al., 2011). However, other studies have failed to find a change in myonuclei number with atrophy, and loss of satellite cells during disuse is not a consistent finding. For example, satellite cell number appeared to be quite stable after acute disuse including 14 days of immobilization in young healthy men (Snijders et al., 2014b), or 28 days of bed rest in middle aged men (Brooks et al., 2010), whereas in another study, 14 days of immobilization resulted in a loss of satellite cells in older humans (Suetta et al., 2013). Severe disuse atrophy as characterized by spinal cord injury has been reported to result in lower satellite cell numbers in both type I and type II fibers (Verdijk et al., 2012). Furthermore, sarcopenia is accompanied by a loss of satellite cells, particularly in type II fibers (Verdijk et al., 2012, 2014) although the reductions in both fiber size and satellite cell number occur relatively slowly. In contrast, rapid declines in muscle mass do not correlate closely with satellite cell numbers (van der Meer et al., 2011b). Nevertheless, satellite cells may have an important role in long-term modulating of muscle fiber size including disuse atrophy, and sarcopenia, but there is evidence to suggest that such changes in myonuclear number may not determine the immediate changes in muscle fiber size (van der Meer et al., 2011a,b). Nevertheless, the age of the host and the severity or type of disuse, likely helps to determine whether satellite cells survive, the time course of any changes in satellite cell number, or how they are able to respond to additional stressors.

Although proliferation of satellite cells is critical to muscle regeneration after an injury, proliferation of satellite cells without adequate differentiation does not improve muscle mass. For example, acute satellite cell proliferation has been reported in response to spinal muscular atrophy-induced muscle denervation (Martinez-Hernandez et al., 2014) presumably as an attempt to increase the nuclear population for elevating transcriptional signaling, yet, the atrophy signaling predominates the muscle, and the net result is that denervated muscles become smaller, even with an acute increase in satellite cells. Thus, effective strategies to combat sarcopenia and accelerated muscle loss in aging should evaluate the effects of interventions on both proliferation and differentiation of satellite cells and their daughter cells.

## Modulation of Satellite Cell Function in Sarcopenia via Nutraceuticals

It is clear that the nutritional status of a host affects the potential for satellite cell proliferation and differentiation to occur (Halevy et al., 2000; Powell et al., 2013, 2014; Harthan et al., 2014). For example, food restriction in birds post-hatch has been shown to reduce muscle mass accumulation with increased fat deposition and necrosis (Velleman et al., 2010) as a result of a decrease in satellite cell mitotic activity (Mozdziak et al., 2002; Halevy et al., 2003). However, the mechanisms by which nutritional interventions regulate satellite cell function are less well defined. One possibility is that the systemic diffusion of nutritional compounds and nutraceuticals from the blood (presumably diffusion from capillaries throughout the muscle) changes the satellite cell environment or “niche.” The area that is enclosed between the basal lamina and sarcolemma of a muscle fiber houses the satellite cell, but this same space provides an insulated environment in which the satellite cell exists (Lander et al., 2012; Bentzinger et al., 2013; Montarras et al., 2013). Presumably this niche maintains the satellite cells in a quiescent state. It is likely that the metabolic milieu of the satellite cell niche differs from the muscle fiber and/or the extracellular space that surrounds the fibers. Although speculative, it is possible that nutraceuticals diffuse from the systemic circulation (i.e., capillaries) and pass through the basal lamina membrane barrier to the satellite cell niche to change its metabolic composition. This idea would be consistent with the hypothesis that satellite cell function can be regulated via changes to the niche environment (Cosgrove et al., 2009; Chakkalakal and Brack, 2012; Chakkalakal et al., 2012; Gilbert et al., 2012). Although it is not clear if the nutraceuticals can directly activate satellite cells to move them from a quiescent to an active state, it is clear that if they have begun a proliferative cycle, that several nutraceuticals can enhance their function in responses to various stimuli superimposed on sarcopenia. However, the evidence suggests that diffusion of the nutraceutical into the satellite cell niche could “prime” the satellite cell, so that once it experienced the appropriate chemical and/or mechanical and/or electrical signals for proliferation, would increase the extent of proliferation in these cells (Hao et al., 2011; Ryan et al., 2011; Alway et al., 2013; Bennett et al., 2013).

It is further feasible that nutraceuticals can act indirectly on satellite cells by modulating or directly suppressing the effects of ROS, or increasing antioxidant production. Either of these possibilities would result in lowering the impact of ROS damage on satellite cells.

Another possibility is that rather than affecting the satellite cell niche, the nutraceutical modulates satellite cell behavior after these cells have left their niche, or perhaps the niche environment is lost because the basal lamina or sarcolemma has been damaged. In this scenario, the cellular milieu containing the nutraceutical mixes with the satellite cell niche and then promotes enhanced proliferation of activated satellite cells. Again this could occur from a direct effect on satellite cells (e.g., epigenetic changes to satellite cells) and/or indirectly via suppressing the effects of ROS on satellite cells. Future studies are required to determine which nutritional interventions change the contents of the satellite cell niche, or if rather, the nutraceuticals have a direct effect on satellite cells that is independent from the niche environment.

In addition to the local satellite cell niche milieu, satellite cell function appears to have an interaction with the fiber that it is attached to. There are more satellite cells that are associated with fibers that are predominantly oxidative (slow, type I fibers), as compared with fibers that rely primarily on glycolysis (fast, type II fibers) (Schmalbruch and Hellhammer, 1977; Putman et al., 2001; Brack et al., 2005; Christov et al., 2007). Nutraceuticals may have differential effects on satellite cells in predominately type II vs. type I fibers. For example, proliferation of satellite cells was increased in plantaris (predominately type II fibers) muscles from old rats that were reloaded after hindlimb suspension following treatment with HMB (Alway et al., 2013) or EGCg (Alway et al., 2014a), whereas satellite cells were elevated in both plantaris and soleus (predominately type I fibers) muscles of old rats that were treated with green tea extract under these conditions (Alway et al., 2014b). As nutraceutical treatments do not appear to have a marked impact on slowing accelerated muscle wasting in sarcopenia (Table 1), but instead appears to be more effective in rehabilitating muscles after a period of disuse (Table 2), we suspect that this treatment strategy modulates and does not activate the satellite cells, as we would not anticipate a high level of satellite cell proliferation during periods of long-term disuse.

**Table 1:** **Summary of nutraceutical effect on apoptosis and muscle function in aging muscle during forced disuse**.

Nutraceutical	Treatment	Apoptotic signaling	Fiber area	Muscle mass	Maximal force	Reference
HMB	HLS	↓(100–600%)	↑(22%)	→	→	Hao et al. (2011)
EGCg	HLS	↓(25–30%)	↑(21%)	→	→	Alway et al. (2014a)
Resveratrol	HLS	→	ND	↑(14%)	↑(14%)	Jackson et al. (2010)
Resveratrol	HLS	→	→	→	ND	Bennett et al. (2013)
Green Tea Catechins	HLS	ND	ND	→	↑(10%)	Ota et al. (2011)
Green Tea Catechins	HLS		↑(10%)	↑(7%)	→	Alway et al. (2014b)

*The arrow indicates the increase (*p* ≤ 0.05), decrease (*p* ≤ 0.05), or no difference (*p* ≥ 0.05) between the vehicle vs. nutraceutical. The percent difference from vehicle treatment is indicated in parenthesis*.

**Table 2:** **Summary of nutraceutical effect on satellite cell function, apoptosis and muscle function in growing/regenerating aged muscle**.

Nutraceutical	Treatment	Satellite cell proliferation	Apoptotic signaling	Muscle mass	Fiber area	Maximal force	Reference
HMB	Reloading after HLS	↑(3%)	ND	↑(6%)	↑(12%)	→	Alway et al. (2013)
HMB	Reloading after HLS	ND	↓(70–100%)	↑(35%)	↑(55%)	↑(15%)	Hao et al. (2011)
HMB	Cell culture	↑(250%)	↓(30–70%)	ND	ND	ND	Kornasio et al. (2009)
EGCg	Reloading after HLS	↑(3%)	↓(23–50%)	↑(14%)	↑(36%)	↑(20%)	Alway et al. (2014a)
Green Tea Catechins	Acute downhill running	ND	ND	→	ND	↑(100%)	Haramizu et al. (2013)
Green Tea Catechins	Reloading after HLS	↑(17%)	↓(36–50%)	→	↑(13%)	↑(25%)	Alway et al. (2014b)
Resveratrol	Aging	ND	→	→	ND	→	Jackson et al. (2011)
Resveratrol	Reloading after HLS	→	↓(0–29%)	↑(10%)	↑(28–45%)	ND	Bennett et al. (2013)

*The arrow indicates the increase (*p* ≤ 0.05), decrease (*p* ≤ 0.05), or no difference (*p* ≥ 0.05) between the vehicle vs. nutraceutical. The percent difference from vehicle treatment is indicated in parenthesis)*.

It is not known if the nutraceutical mediated improvement in satellite cell proliferation especially during rehabilitative efforts in sarcopenic muscle, was due to direct signaling from the fiber to the satellite cell niche or the satellite cells themselves, and it is not known how the satellite cell niche, fiber type, or extracellular matrix signaling might influence satellite cell function in response to skeletal muscle regeneration or hypertrophic growth in sarcopenic muscles from old hosts. These questions should be the focus of future studies as this information will be helpful in planning strategies that might improve muscle repair and slow the progression of sarcopenia. Furthermore, understanding the effect of nutraceuticals on satellite cells in a fiber-type specific fashion is important, because the satellite cells in fast muscles appear to be more vulnerable to dysfunction in aging and show a reduction in total satellite cell numbers (Verdijk et al., 2007, 2014) as compared to satellite cells associated with type I fibers. Nevertheless, while slow oxidative muscles seem to be better preserved than the fast fibers in sarcopenic muscles (Deschenes et al., 2013; Purves-Smith et al., 2014), the impact of nutraceuticals on type I fiber associated satellite cells should not be ignored, because this preservation of type I fiber size and function might be lost in the very old, when sarcopenia becomes very severe (Purves-Smith et al., 2014).

### Resveratrol

#### Resveratrol and satellite cell function

Within the past decade, sirtuin 1 (Sirt1), a NAD^+^ dependent deacetylase, has been identified as an important metabolic regulator of skeletal muscle gene expression (Fulco et al., 2003). Specifically, elevated Sirt1 activity has been shown to increase proliferation of satellite cells (Rathbone et al., 2009). Furthermore, Sirt1 has been reported to inhibit the differentiation of mouse C2C12 myoblasts (an *in vitro* model of activated satellite cells), and reduce the expression of myogenin an important regulator for satellite cell (daughter cell) differentiation (Fulco et al., 2003; Vinciguerra et al., 2010). This suggests that Sirt1 could have a role of delaying differentiation and therefore prolonging or enhancing proliferation of satellite cells in response to a growth stimulus. Furthermore, reduced nutrient availability inhibits C2C12 myoblast differentiation in a Sirt1 dependent manner (Fulco et al., 2008). Interestingly, the NAD^+^ salvage enzyme nicotinamide phosphoribosyltransferase was found to mediate the effects of nutrient (glucose) deprivation on myogenic differentiation *in vitro* (Fulco et al., 2008). However, to this point, it is not clear if Sirt1 has a direct or an indirect role in mediating satellite cell proliferation or differentiation *in vivo* in aged/sarcopenic muscles. Nevertheless, we have some clues through other nutritional based intervention studies that suggest that resveratrol, through Sirt1 has a direct effect on regulating satellite cell function in aging. For example, resveratrol, a Sirt1 activator that was given to old rats during a period of recovery following hindlimb suspension had a modest improvement in satellite cell proliferation in hindlimb muscles in response to cage ambulation that followed period of muscle disuse as compared to a vehicle control treatment (Bennett et al., 2013). It is also possible that resveratrol has multiple effects in aged muscles. For example, it appears to also have a more profound protective effect in aging by buffering high levels of oxidative stress which is amplified in old animals during periods of muscle disuse or loading (Jackson et al., 2010; Ryan et al., 2010; Joseph et al., 2013; Durbin et al., 2014). Furthermore, although a constant long-term consumption of resveratrol does not eliminate sarcopenia (Jackson et al., 2011), it is possible that increasing the dose of resveratrol as the animal ages, to better counter the increasing ROS accumulation (and ROS increases with greater age) might have had a different outcome. Nevertheless, together the data suggest that resveratrol might lower excessively high ROS levels, and this would be expected to improve satellite cell function and/or prevent loss of some of the activated satellite cells in responses to stressors (e.g., loading), that otherwise might be lost (destroyed) in a very high ROS environment such as aging and loading, although it probably has modest effects on quiescent satellite cells that are attached to sarcopenic muscles. It is noteworthy that increased levels of Sirt1 have been reported in satellite cells isolated from old rats, although the significance of this is not clear (Machida and Booth, 2004). Thus, it is possible that when Sirt1 was elevated in satellite cells of old animals, it may not have been active and therefore unable to produce the anticipated benefits that have been associated with Sirt1 in aged muscles. This is likely because activated Sirt1 has been shown to directly induce proliferation of satellite cells (Rathbone et al., 2009).

#### Resveratrol – a link to inflammation mediated satellite cell function?

In addition to its role in metabolism-regulation of satellite cells, resveratrol, through Sirt1 deacetylates and activates PGC1α, which in turn activates transcription factors like the farsenoid x receptor (FXR), PPARα, and PPARδ, which have anti-inflammatory effects (Galuppo et al., 2010; Xu et al., 2012). Thus, it is possible that PGC1α’s role in muscle regeneration may be to signal the end of the inflammation period and begin the period of regeneration (satellite cell proliferation and/or differentiation).

Though the role of inflammation in muscle regeneration is currently unclear, the current thought is that early inflammation inhibits muscle regeneration, so that necrotic and damaged cells may be cleared from the injury site, while the later response, such as the arrival of macrophages, has a stimulating effect on muscle repair. Furthermore, macrophages increase the proliferation rate of satellite cells, while depletion of macrophages after a muscle injury has been shown to inhibit satellite cell function to reduce the rate of muscle regeneration (Tidball, 2005; Tidball and Wehling-Henricks, 2007). Thus, understanding PGC1α’s connection to inflammation (Westerbacka et al., 2007) and regulation of mitochondrial biogenesis and satellite cell function through resveratrol or other nutraceuticals, may help to further elucidate its role in the inflammatory response period that is associated with muscle regeneration in aging.

### Epigallocatechin gallate and satellite cells

One of the most abundant catechins in green tea is epigallocatechin-3-gallate (EGCg), which has strong antioxidant and anti-inflammatory properties. EGCg is believed to be responsible for most of the health benefits linked to green tea. Both disuse and reloading which accelerate muscle loss in sarcopenia, greatly increase the oxidative stress in the affected muscles of old animals (Andrianjafiniony et al., 2010; Jackson et al., 2010; Pellegrino et al., 2011). Reducing the high basal levels of oxidative stress in aging could potentially attenuate muscle mass decrement that occurs in response to disuse conditions and/or improve muscle recovery during reloading after disuse in aging (Jackson et al., 2010). Recent data suggest that oxidative stress is reduced both in cultured cells (Casanova et al., 2014) and after eccentric exercise upon supplementation with green tea catechins (Haramizu et al., 2011). Furthermore, green tea catechins reduce the decrement in soleus muscle force during a period of hindlimb suspension in mice (Ota et al., 2011). In addition, EGCg has been shown to reduce protein degradation in culture (Mirza et al., 2014). We have recently shown that activation of satellite cells as shown by labeling with the thymidine analog 5-bromo-2-deoxyuridine (BrdU), was significantly greater in reloaded muscles of old rats after a 14 days of hindlimb suspension muscle disuse as compared to muscles from vehicle-treated old animals (Alway et al., 2014a). Interestingly, this appeared to be a fiber or muscle-specific effect on satellite cell proliferation, because while 14 days of reloading increased BrdU labeled nuclei in the plantaris from EGCg treated muscles (7.4%) compared to vehicle-treated animals (6.3%), EGCg did not improve satellite cell activation in the soleus muscle of reloaded animals. Using green tea extract that contained approximately 50% EGCg, we found that satellite cell proliferation and differentiation of the satellite cell daughter cells were both increased in muscles of old rats during reloading after 14 days of hindlimb suspension (Alway et al., 2014b). Moreover, data from old mice and humans that were fed EGCg for 7 days, showed improved markers for satellite cell activation (Myf5, MyoD) (Gutierrez-Salmean et al., 2014). However, EGCg treatment also reduced anabolic suppressor proteins (e.g., myostatin) (Gutierrez-Salmean et al., 2014), so it is more difficult to tell if EGCg provides a direct or indirect effect on satellite cell function. Nevertheless, together these results show that EGCg and perhaps other catechins contained in green tea were effective in improving satellite cell proliferation. We speculate that having more available satellite cell derived daughter nuclei supported the adaptation for greater muscle cross sectional area and this improved the recovery of muscle mass following disuse in sarcopenic rat muscles (Alway et al., 2014a).

### HMB regulation of satellite cells in sarcopenia

The leucine metabolite, β-hydroxy-β-methylbutyrate (HMB) has been shown to improve satellite cell proliferation (Moore et al., 2005), reduce protein catabolism during disease, reduce muscle loss during disuse, and promote skeletal muscle hypertrophy in response to loading exercise (Wilson et al., 2008; Holecek et al., 2009; Aversa et al., 2011). We have previously shown that HMB could improve muscle recovery in old rats with sarcopenia that had been subjected to unloading, in part via an increase in satellite cell proliferation and a reduction of nuclear apoptosis (Hao et al., 2011). HMB also has been shown to have direct effects on proliferation of myoblasts *in vitro* (Kornasio et al., 2009), although, its efficacy on satellite cell activation has not previously been evaluated *in vivo* in aged immobilized animals. We found that HMB-treatment in old rats during reloading after forced disuse resulted in a significantly greater (*p* < 0.01) level of BrdU positive satellite cells in plantaris muscle cross sections of aged HMB-treated animals (9.1% of total myonuclei) as compared to the animals in the vehicle group (6.1% of total myonuclei) (Alway et al., 2013). This was confirmed by a greater percentage of Pax7^+^ and MyoD^+^ myonuclei (derived from satellite cells) relative to the total myonuclear pool in reloaded plantaris muscles as compared to reloaded muscles from vehicle-treated old animals (Alway et al., 2013). Thus, the mechanism of action through which HMB is responsible for enhancing muscle recovery following extended disuse in sarcopenic muscles of old rats appears to be at least in part, via increased proliferation of muscle satellite cells in fast twitch plantaris muscles of aged animals. However, the enhancement of satellite cell proliferation by HMB is not a universal finding. For example, older women who were fed HMB for six days during a period of resistance training to load their skeletal muscles had a ~100% increase in satellite cell numbers, but HMB did not increase satellite cell proliferation further over resistance training alone (Kim et al., 2012). Additional work is needed to determine if the beneficial effects of HMB on satellite cell function will be observed in older humans if the dose or duration of HMB is altered.

## Regulation of Nuclear Death Signals by Nutraceuticals

Satellite cells that are isolated from sarcopenic muscles from old rodents and humans have a greater propensity for apoptosis and greater levels of apoptotic signaling proteins (Fulle et al., 2012, 2013). Not only can apoptosis signaling target mature post mitotic nuclei for elimination, but satellite cells and their daughter cells that are activated as part of a hypertrophic adaptation to a loading stimulus, can be targets for elimination as well (Alway and Siu, 2008). Satellite cell number decreases with increased aging (Snow, 1977; Verdijk et al., 2014), and one possibility to explain this reduction in satellite cells is due to an increased susceptibility to nuclear apoptosis in aging and therefore, this may contribute to sarcopenia (Leeuwenburgh, 2003; Pistilli et al., 2006; Adhihetty et al., 2008, 2009; Ljubicic et al., 2009; Alway et al., 2011; Quadrilatero et al., 2011; Marzetti et al., 2012; Calvani et al., 2013). Furthermore, the pro-apoptotic protein Bax, is increased in satellite cells of old rats (Krajnak et al., 2006) and this leads to accelerated muscle loss in sarcopenic muscles via apoptosis (Dupont-Versteegden et al., 2006; Pistilli et al., 2006; Alway et al., 2014a). However, signaling for apoptosis is reduced and more satellite cells (e.g., Pax7/MyoD^+^ cells) survive during rehabilitation after disuse in aged rat muscles that are treated with HMB (Hao et al., 2011; Alway et al., 2013), EGCg (Alway et al., 2014a), or green tea catechins (Alway et al., 2014b) as compared to a control treatment. In contrast, resveratrol fed animals had lower levels of oxidative stress, but only modest changes in apoptotic signaling (Jackson et al., 2010; Bennett et al., 2013) as compared to control animals. Although the mechanism(s) by which nutraceuticals impact satellite cell function, including reducing pro-apoptotic targeting of satellite cells, is likely to be complex. Part of the improvement in apoptotic signaling in activated satellite cells may be due to an upregulation of antioxidants and a reduction of oxidative stress and/or inflammation after nutraceutical treatments including resveratrol (Jackson et al., 2010, 2011; Ryan et al., 2010) and green tea catechins (Ota et al., 2011; Wang et al., 2011; Andrade and Assuncao, 2012; Wu et al., 2012; Haramizu et al., 2013). Given the propensity for apoptosis to occur in satellite cells isolated from old hosts including humans (Fulle et al., 2012, 2013), further investigations into the potential for nutraceuticals to improve satellite cell function in aging are warranted. Together these data support the idea that reducing the systemic (and perhaps also the satellite cell niche) signaling for apoptosis, may promote better survival of satellite cells and their daughter cells in muscles of old animals, and this may contribute to improved muscle recovery after periods of disuse (e.g., hospitalization) and reduce the effects of sarcopenia in the elderly.

## Conclusion

**Figure 2:**
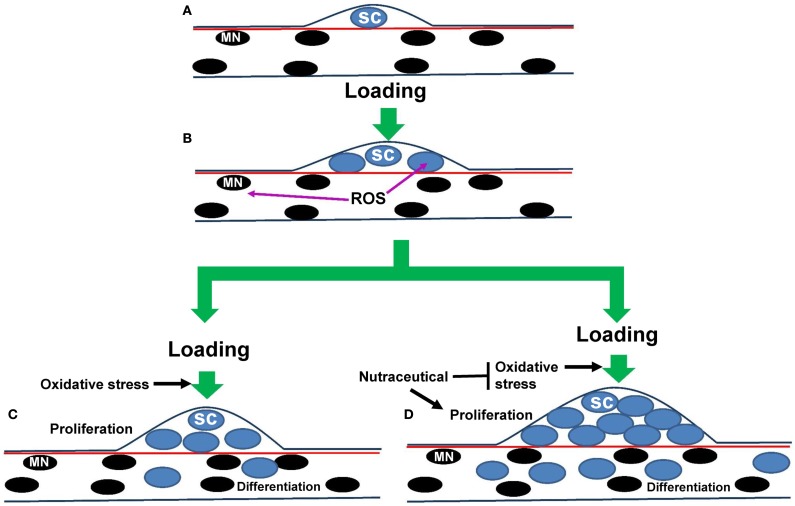
**Hypothetical model for nutraceutical effects on satellite cell proliferation in sarcopenia during muscle loading**. **(A)** A muscle fiber is illustrated that shows myonuclei (black) and a single satellite cell (blue) that is positioned between the basal lamina (dark blue line) and the sarcolemma (red line). **(B)** Loading of aged muscles results in proliferation of the original satellite cell nuclei, but it also increases reactive oxygen species (ROS) accumulation, in part as a result of aging-induced changes in mitochondria. High levels of ROS in aged muscles can trigger apoptotic pathways which presumably have the potential to eliminate the targeted nuclei and activated satellite cells. **(C)** After proliferation, some of the daughter cells will migrate into the adult fiber and differentiate to support growth. However, as satellite cells from old animals tend to have a greater propensity (and maybe sensitivity) to apoptosis-induced cell death, not all of the proliferating satellite cells including those that differentiate survive and some will remain in the position of satellite cells to replenish the satellite cell population. **(D)** Nutraceuticals resveratrol, EGCg, and green tea appear to enhance the proliferation of satellite cells in response to an activating stimulus (e.g., muscle loading), perhaps in part by reducing ROS and in some cases improving mitochondrial number or function. This provides a higher number of surviving daughter cells that are able to migrate into the adjacent fiber, to presumably support growth and/or repair of skeletal muscle fibers in aging. It is also possible that the proliferated protege from satellite cells could signal other cell types or change the muscle fiber niche that would be conducive for hypertrophy or slowing muscle losses in sarcopenia.

Although the satellite cell has been identified and studied for more than a half of a century (Mauro, 1961), there is still much that we do not know about this unique muscle stem cell in aging. In general, there is a reduction in satellite cell number and function that occurs with aging, especially in type II fibers, but this does not seem to be due to increased DNA damage in these cells (Cousin et al., 2013). Nevertheless, it is clear that satellite cell proliferation and differentiation contributes to a greater myonuclear pool. Improving satellite cell proliferation occurs especially in fast muscles of aged animals provided supplemental HMB, EGCg, resveratrol or green tea, and a greater number of satellite cell derived nuclei should provide a greater potential for transcriptional and translational control for improving regeneration in aged muscles (Figure 2). One possibility is that the nutraceuticals act to buffer the high levels of ROS in aging muscles of old animals. Nutraceuticals may also reduce the level of oxidative stress that is elevated in aging muscles in response to loading or disuse. The less oxidative environment may encourage the survival of more of the activated satellite cells so that they can participate in muscle repair. Additionally, as EGCg has been shown to improve muscle function following a nerve crush injury (Renno et al., 2012), it would be interesting to know in future studies if catechins or other nutraceuticals could delay or suppress age-associated denervation. Furthermore, nutritionally regulated reductions in the potential for death signals (e.g., apoptosis) to eliminate satellite cell progeny that have migrated inside a muscle fiber should also improve the potential for transcriptional and translational regulation of muscle fiber regeneration or repair in aging. Thus, nutraceuticals appear to have the potential to regulate satellite cell function, and in doing so, impact skeletal muscle regeneration, particularly during rehabilitative efforts that follow a period of disuse in aged animals (Table 2). Unfortunately, nutraceuticals do not appear to have profound effects on slowing accelerated loss in sarcopenic muscles (Table 1).

Clinical trials in humans are warranted to determine if these or other nutraceuticals, will similarly improve muscle recovery following bed rest or other conditions of muscle loss in aging as observed in rodents. However, it has only been recent that we have begun to appreciate the potential links between nutrition and metabolism and satellite cell function in health and disease. In the context of treating sarcopenia, it is important to note that not all changes in diet that might slow muscle loss, necessarily affect satellite cell function. For example, dietary protein intake alone does not modulate the post-exercise increase in satellite cell content but instead, it modifies myostatin expression in skeletal muscle tissue, which contributes to the increase in protein accretion after acute exercise (Snijders et al., 2014a). Thus, it is important to distinguish between satellite cell and non-satellite cell functions of nutraceuticals in sarcopenic muscles of the aging hosts. Understanding the nutritional regulation of satellite cell function appears to be a potentially promising avenue for identifying strategies to reduce muscle wasting in sarcopenia, and to improve the recovery of muscle that is lost during a period of disuse in aged humans.

## Conflict of Interest Statement

The authors declare that the research was conducted in the absence of any commercial or financial relationships that could be construed as a potential conflict of interest.
